# Automation in Dentistry with Mechanical Drills and Lasers for Implant Osteotomy: A Narrative-Scoping Review

**DOI:** 10.3390/dj12010008

**Published:** 2023-12-29

**Authors:** Gopala Krishna Ganta, Rodrigo Crespo Mosca, Ridham Varsani, Venkata Ramana Murthy, Kamala Cheruvu, Michael Lu, Praveen R. Arany

**Affiliations:** 1 Oral Biology, Biomedical Engineering & Surgery, University at Buffalo, Buffalo, NY 14214, USA; 2 Intercare Community Health Network, Bangor, MI 49013, USA; 3 Department of Maxillofacial Surgery, Anil Nirukonda Dental College, Visakhapatnam 531162, India; 4 Department of Orthodontics, Gandhi Institute of Technology and Management Dental College, Visakhapatnam 530045, India

**Keywords:** automation, robotics, implants, osteotomy, lasers

## Abstract

The popularity of implants is increasing with the aging population requiring oral–dental rehabilitation. There are several critical steps in the implant workflow, including case selection, implant design, surgical procedure, biological tissue responses, and functional restoration. Among these steps, surgical osteotomy procedures are a crucial determinant of clinical success. This brief review was aimed at outlining the current state of the field in automation-assisted implant surgical osteotomy technologies. A broad search of the literature was performed to identify current literature. The results are outlined in three broad categories: semi-automated static (image-guided) or dynamic (navigation-assisted) systems, and fully-automated robotic systems. As well as the current mechanical rotary approaches, the literature supporting the use of lasers in further refinement of these approaches is reviewed. The advantages and limitations of adopting autonomous technologies in practical clinical dental practices are discussed. In summary, advances in clinical technologies enable improved precision and efficacious clinical outcomes with implant dentistry. Hard-tissue lasers offer further advancements in precision, improved biological responses, and favorable clinical outcomes that require further investigation.

## 1. Introduction

The increase in human longevity has resulted in a larger aging population with increased demand for dental services. Owing to the rising occurrence of edentulism, a larger age group in the population, in addition to geriatrics, is actively seeking implant treatments [1]. The absence of teeth can result in more than just esthetic and functional limitations, as inadequate nutrition and subsequent declines in overall health have been correlated [2,3,4]. Such negative outcomes increasingly correlate with several systemic diseases. Implants offer an expedient option for prosthetic rehabilitation in edentulous patients. Since Brånemark first described the utility of dental implants, they have undergone significant development, including enhancements to their form, surface quality, procedural techniques, and functional diversifications.

A successful implant placement relies on effective treatment-planning that significantly hinges on the implant osteotomy’s location, design, and technique [5,6,7]. The dental-implant-placement process can be categorized into three phases: the planning phase, the surgical phase, and the rehabilitation or prosthesis phase [8]. Planning for dental implants is intricate and involves considering multiple factors. These factors encompass a range of aspects such as a patient’s age, physiological health, and habits, as well as aesthetic concerns, bone density availability, implant design variations, and ultimate prosthesis placement. Subsequently, a template design is created, and a mock implant-placement procedure is conducted to confirm the selected parameters for use on the actual day of surgery. This rigorous planning phase precedes the surgical stage of implantation. Several of these procedural steps, from imaging, to planning with computer-assisted design (CAD), to 3D additive printing for surgical splints to guide surgery, and even custom-implant fabrications, have been improved with digital workflow [9].

The process of implant surgery requires careful consideration and decision-making at multiple stages. An experienced practitioner must evaluate the situation thoroughly and be prepared to deviate from pre-established protocols if necessary. As the demand for dental implants rises, healthcare providers face the challenge of ensuring optimal treatment planning and delivery while dealing with constraints such as limited time and fatigue, which can affect surgical outcomes. This crisis is aggravated by the scarcity of qualified licensed professionals to perform implant-placement procedures, as well as their restricted geographical reach, limited availability, and higher professional fees. To address these challenges and ensure consistent treatment quality among all patients, automation using robotics for implant placement has gained popularity. The technologies supporting robotic implant placement have evolved over the years, resulting in significant clinical benefits. Initially used only as a guiding mechanism, current technology includes assistive measures, semi-automatic systems, and, most recently, fully automated systems [9].

## 2. Search Methodology and Summary of Results

**Figure 1:**
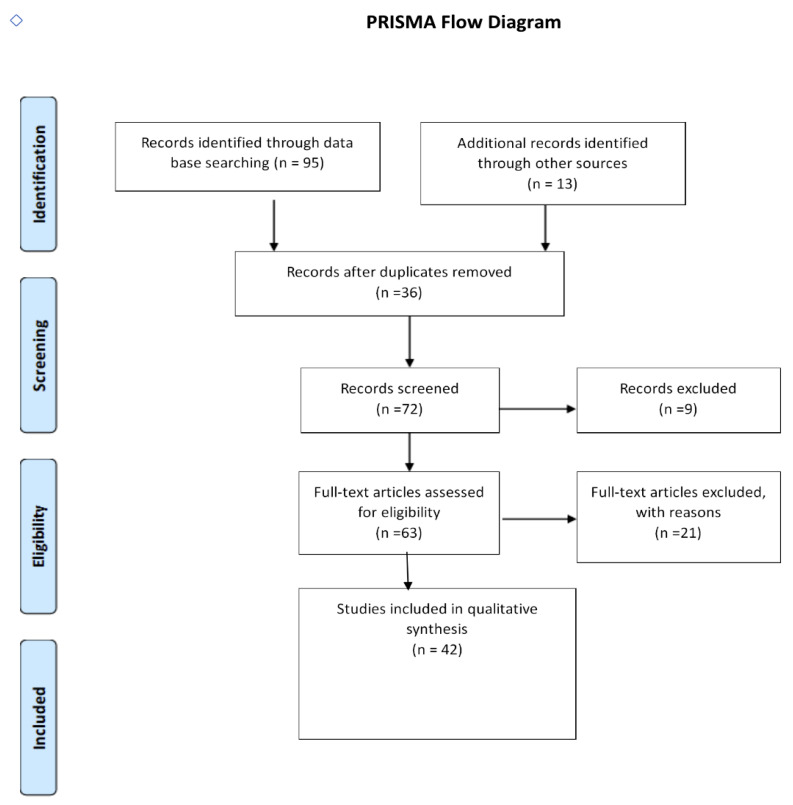
Workflow outlining the literature search and review performed in this work. Although this workflow simulated a routine systematic review process (PICO), a narrative and scoping review is presented due to a lack of relevant literature available for analysis on this subject.

This article examines the currently available technologies of dental implant surgery. We carried out a search of Medline, PubMed, Scopus, and Cochrane databases along with a broad internet (Google) search to review the literature using search terms ‘robotics’, ‘lasers’, ‘osteotomy’, ‘automation’, ‘laser osteotomy’, ‘laser-guided implant placement’, and ‘laser implant osteotomy’, restricted to the past 15 years (Figure 1). The initial search resulted in a total of 108 articles of which 36 were found to be duplicates and 9 were in a language other than English and excluded. Full-text articles were downloaded, and after filtering, 42 articles were finally chosen for inclusion in this review. The results in each category are summarized below, with a broad introductory section.

## 3. Common Features of Robotic or Automated Implant Surgery

**Figure 2:**
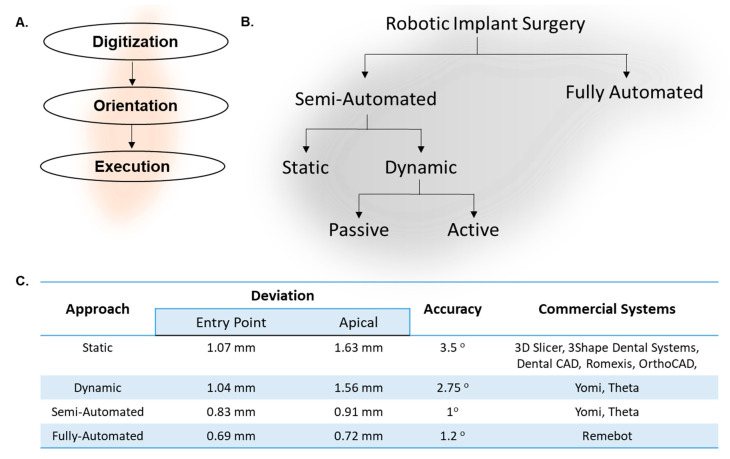
Automated implant osteotomy. (**A**) Procedural steps in implant osteotomy automation workflow. (**B**) Broad categorization of various approaches for discrete levels of automation. (**C**) Performance characteristics of various automated implant osteotomy approaches in the literature.

Automation for implant surgery involves a conglomeration of technologies that deal with image-processing software, navigation software, and optical-guided tracking systems [10,11,12,13]. First, the digitization for the treatment-planning software outlines the strategic placement of the implant to ensure there is no harm to crucial anatomical structures obstructing its ultimate position (Figure 2A) [14]. These software technologies utilize machine-learning approaches to continuously scrutinize and distinguish the essential components from the radiographs, usually cone-beam computed tomography (CBCT) and intraoral digital imaging [11]. Based on the statistical information fed to the machine-learning algorithm, it can identify imaging errors like shrinkage, magnification, image noise, and distortions to identify the landmarks accurately. The software suggests all the possible implant configurations like implant size, angulation, positioning, and aggressiveness of the threads based on the quality and quantity of bone.

The next step is orientation, involving registration and navigation of the operator or robotic arms by taking inputs from the fiducial markers used in the surgical setup. Fiducial markers are specialized devices that give the system feedback on the positioning of all the mobile surgical heads in the surgical field. They determine the relative distances and boundaries of movements of the robotic arm within the surgical site as per the treatment plan in real time [10,11]. The two major fiducial technologies include electromagnetic and optically-guided tracking systems [15]. Both systems have a few limitations in that the electromagnetic fiducials could be distorted by the presence of ferromagnetic substances in the surgical field. The optically-guided fiducials required a straight line (line-of-sight) between the camera and the fiducial at all times for accuracy. These fiducials need to be calibrated and overlap with the existing CBCT and digital data, so the navigational system can assist with either guided semi-automated or fully automated movements [12,15]. To ensure the precision and accuracy of the surgical process, a trial placement of an artificial implant on 3D-printed models is strongly suggested. In addition to clinical accuracy, this simulation also serves to detect any unexpected movements in the robotic arms and inaccessible sites prior to surgery [11,14].

The navigation software utilizes relative three-axis template systems guided by an optical tracking system (OTS) in real time which can identify the fiducials and bring movements with the help of robotic arms with reference to their relative positions [10,12]. Through meticulous programming, the system’s algorithms are capable of determining and initiating precise robotic-arm movements required for positioning surgical instruments accurately. The tip of the osteotomy instrument will then initiate the drill and sequentially create an osteotomy for the implant starting with a pilot drill and proceeding sequentially to the expected diameter and depth with the specified angulation, as per the approved surgical plan. The degree of freedom and the torque of the robot and the motors, respectively, resonate with the precision of the osteotomy and the planned procedure, so higher specifications will produce better results [12].

The final step in this workflow involves the execution of surgical procedures. Current technologies can be broadly bifurcated into two major categories based on the level of automation and involvement of a clinical operator (Figure 2B). The use of image-guided, navigation-assisted technologies enables the operator to improve their surgical implant outcomes by improving spatial orientation and achieving a heightened level of parallelism, particularly when multiple implants are required within a single jaw [10,12]. These technologies are versatile and compatible with various implant systems available in the market. This technique eliminates the need for surgical guides, maintaining accuracy and reproducibility of outcomes, enabling surgeons to operate in critical areas with limited visibility. There are a few drawbacks like the costs of equipment, accessibility in patients with limited mouth opening, and requiring a drill extender for operating in the posterior regions of the oral cavity. While this procedure can improve the accuracy of placement of the implant, the inherent disadvantages of drills, like osteonecrosis of the adjacent bone due to the heat dissipation of mechanical burs and the micro-cracks induced due to the friction and vibrations, should be assessed [16,17,18,19]. In contrast to this assisted approach, there are recent innovations in fully-automated robotic systems that show striking promise, as evidenced by their accuracy (Figure 2C), that are briefly reviewed in the following sections.

## 4. Static Approach: Computer-Guided Implant Surgery

**Figure 3:**
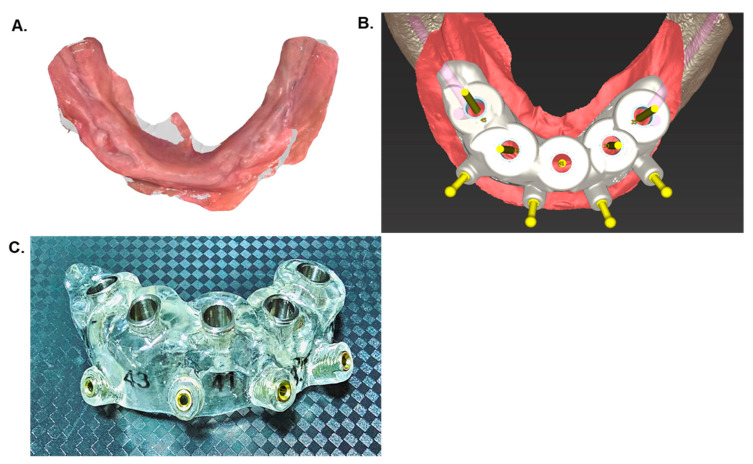
Digital planning (**A**). Digital planning for dental implants was conducted using 3D modeling imaging on an IOS (iTero^®^—Align Technology, Inc., San Jose, CA, USA) platform. (**B**). Computer-aided design (CAD) was utilized to plan a digital surgical guide for the edentulous mandible, incorporating five implants and four mini-implants for anchorage. (**C**). The surgical guide, printed after the virtual planning phase, is now prepared for use.

The process of computer-assisted implant surgery comprises the creation of a guide or template to facilitate the accurate placement of the digitally planned implant based on the patient’s CBCT data. This involves utilizing CAD-CAM or 3D printing techniques to generate the guide [20,21,22]. Notably, these guides can be either tooth-supported, bone-supported, or mucosa-supported [22,23]. The creation of a customized surgical guide for dental implants involves a dual-pronged approach utilizing advanced technologies (Figure 3). This includes intraoral scanner (IOS)-based 3D modeling technologies and the acquisition of CBCT data, significantly enhancing the precision and efficacy of clinical surgery procedures. The 3D scanning process employs an automated multi-image method to capture patient-specific dimensions, shapes, and the structure of all oral anatomy. Employing a handheld wand-like device, the scanning procedure involves emitting a structured light pattern onto the surfaces within the patient’s mouth. This pattern interacts with oral structures, reflecting to the scanner’s camera, which captures these reflected light patterns. Consequently, a detailed record of the teeth and soft tissues’ exact details and contours within the oral cavity is created. The scanner rapidly captures numerous images as it moves around the mouth, compiling and processing them in real time using specialized software. This process generates an intricately detailed and accurate 3D digital model of the patient’s dentition and oral anatomy. Subsequently, the collected data undergo conversion into a digital file format, allowing for convenient viewing, manipulation, and electronic storage [24,25,26,27]. The fusion of CBCT and 3D modeling images, following virtual planning, yields a standard triangle language (STL) file suitable for stereolithography. This file is then utilized in a 3D printer, where acrylic resin is employed to replicate the precise pathway initially designed via computer-aided design (CAD) [28,29].

**Figure 4:**
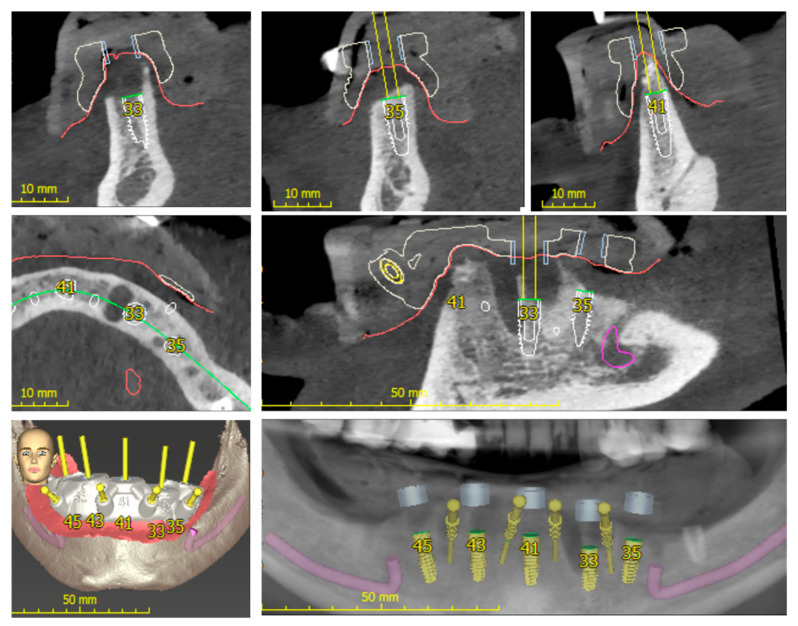
Procedural steps during the digital treatment-planning stage for implant placement. The use of precise implant lengths, based on the key anatomical landmarks and bone volume, is depicted.

The concept of using a sleeve-within-a-sleeve in implant dentistry leads to highly accurate outcomes. This approach employs specific drill sequences that guide the bone’s diameter and orientation, ensuring precision during the surgical procedure (Figure 4). This can lead to a minimally invasive osteotomy, preserving vital structures with accuracy and less operative time. A few limitations of this approach include inherent minor errors of CBCT imaging, mechanical bur-angulation errors, and errors due to the complexity of the clinical procedure. Moreover, intra-operative modifications during surgery are not possible. Meta-regression analysis of the accuracy of this approach noted a mean deviation of 1.07 mm (95% confidence interval, CI: 0.76–1.22 mm) at the entry point and 1.63 mm (95% CI: 1.26–2 mm) at the apex for this protocol. On average, an angular deviation of 3.5° (95% CI: 2.71–3.88) was observed for fully and partially edentulous cases [30].

## 5. Dynamic Approach: Computer-Navigated Implant Surgery (Active or Passive)

**Figure 5:**
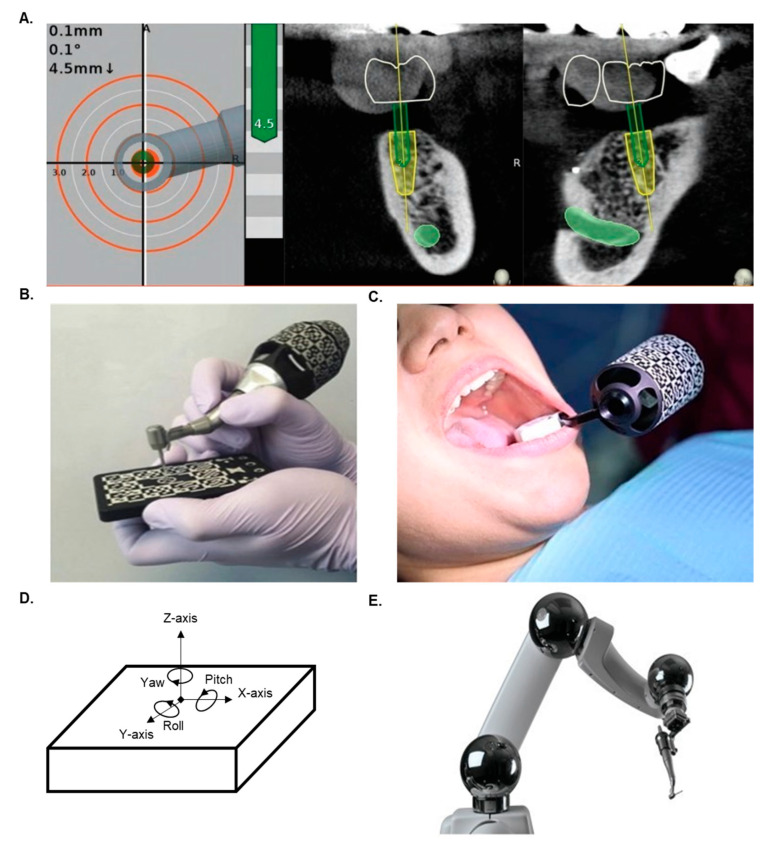
(**A**) Dynamic axial orientation guidance of the head of the handpiece and the bur tip. (**B**) Coordinate registration of the surgical site with a fiduciary to a non-motile site off the site of surgery. (**C**) Coordinate registration for the bur tip and the handpiece with fiduciary before initiation of the procedure-planning software with spatial orientation and anatomical landmarks. (**D**) Visual depiction of the 6-dimensional sensing and rotational degrees of freedom offered by the robotic arms. (**E**) The fully automated YOMI robot with handpiece.

The dynamic approach combines the CBCT data of the patient with the optical bur-tracking navigational software with high accuracy (1 × 1 mm). This includes marking the tissues with the help of fiducials and then identifying the same landmark in the CBCT and overlapping both. The bur-tip orientation can also be captured with the help of a fiducial attached to the handpiece and registered in the system [12,13,31,32,33]. Data about these orientations are fed to the real-time system using a camera or detector to calculate and identify the virtual position of the instruments relative to the CBCT data and the pre-planned implant position (Figure 5A–C) [13,14]. If there are any variations in the bur’s angle or position, a built-in alert system will notify the user. The advantage of this system is that this system bypasses the use of templates or surgical guides by facilitating comfort and ease of operation in patients with limited mouth opening. There are two versions of this system, namely passive and active systems. In the passive form of the system, once the orientation and navigation planning are complete, the system uses this digital roadmap to alert the operator to any deviations of angulation or depth during the procedure. In the active form of the system, unlike the predetermined sleeve-within-a-sleeve model, it utilizes feedback from any patient movements and dynamically adjusts the treatment trajectory to aid the operator in real time (Figure 5D). Meta-regression analysis of the accuracy of this approach noted a mean deviation of 1.04 ± 0.37 at the entry point and 1.56 ± 0.52 mm at the apex for this protocol, and, on average, an angular deviation of 2.74 ± 0.67° was observed for both fully and partially edentulous cases [20,34].

## 6. Robot-Assisted Implant Surgery (Semi-Automated)

A further iteration of the active dynamic approach has been the utilization of a robotic arm to assist the surgeon in placing implants [10,13,20]. These robotic technologies are leveraging the progress in artificial intelligence and machine learning to further enhance precision [12,35]. The inclusion of haptic feedback in the form of either sound or vibration also alerts the surgeon to deviations from the planned surgical osteotomy. During implant surgeries, this system can also serve as a means of modifying the osseous contour intraoperatively [10]. Meta-regression analysis of the accuracy of this approach noted a mean deviation of 0.83 ± 0.55 mm (*p* = 0.04) at the entry point and 0.91 ± 0.56 mm at the apex for this protocol, and an angular deviation of 1 ± 0.48° (*p* < 0.005) for both fully and partially edentulous cases [10,12,13,34].

## 7. Fully-Automated Robotic Implant Surgery

The most sophisticated technology for implant surgery is seen in the evolution of fully automated autonomous planning and implementation of implant placement. One such system has already been made commercially available (Figure 5E) [13,36]. It should be emphasized that the clinical operation of these units still requires a clinical operator’s approval of the planning and execution of the procedure. The procedural workflow is similar to steps described previously involving imaging, treatment planning, registration, navigation, and execution, but the final step occurs in a fully automated manner [11,13,21,36]. Once the registration process is completed, the robotic arm initiates the sequential osteotomy drills as planned [11,36]. Following osteotomy as per the predetermined specifications, the robotic arm will proceed with implant placement. In preliminary studies, the accuracy of this procedure has been determined to have an entry and apical deviation of 0.69 ± 0.15 mm and 0.72 ± 0.16 mm, respectively, with an angular deviation of 1.21 ± 0.54° [11,13].

## 8. Utility of Hard-Tissue Lasers

Laser beams are routinely used in the material-processing industry for tough, resilient materials such as cutting metals and diamonds [37]. As noted above, all current technology adapts the routine rotary mechanical burs for the surgical osteotomy procedure. However, there are recent systems that have been exploring the utility of hard-tissue lasers for implant osteotomy [38]. Interestingly, hard-tissue lasers have been increasingly utilized in orthopedic surgery [37]. However, their clinical utility has been noted to be restricted due to the negative response resulting from reported cases of failure. These limitations have been attributed to a lack of thorough research on laser-tissue surgical interaction, poor understanding of laser parameters, inadequate or lack of training, and inappropriate or poor clinical implementation. However, it is prudent to emphasize a thorough understanding of light–tissue interactions and appropriate laser use has shown tremendous benefits for routine use in dermatology and in ophthalmology, perhaps best exemplified by its daily use in the delicate, exquisitely light-sensitive laser-assisted in situ keratomileusis (LASIK) eye surgery. Thus, in-depth knowledge of laser dosimetry and manner of use is critical for proper clinical applications to attain the best results [17,18,37].

Direct comparison of laser versus conventional mechanical osteotomy for implants appears to be lacking. Nonetheless, there is research on the use of hard-tissue lasers on mineralized tooth tissues, namely enamel and dentin, for tooth cavity preparation [18,39]. Laser cavity preparation has gained popularity as it minimizes bacterial contamination, and could contribute to reduced secondary caries after restoration. Additionally, laser-treated tooth surfaces have been noted to have increased calcium-to-phosphorus ratios that decrease susceptibility to acid dissolution and increase resistance to bacterial enzymes, thus, also aiding in reduced caries incidence. Further, laser cavity preparation eliminates smear layers with a consistency similar to that produced by acid etching. Thus, this forms an ideal surface for composite hybrid adaption as compared with conventional bur preparation [38]. In long-term studies, cavities that were prepared using hard-tissue lasers exhibited superior marginal integrity and ledge configurations compared with those created through traditional cavity-preparation methods [40]. Moreover, laser surgery offers improved patient comfort due to the fusion of dentinal tubules during cavity preparation as well as the lack of mechanical vibration evident with burs, promoting reduced anesthesia and post-surgical discomfort and pain.

For bone ablation in implant osteotomy, the laser ablative surgical processes directly transfer laser energy to the water and hydroxyapatite crystals in the mineralized tissues [17,18,41]. This results in direct ablation or vaporization of the tissue with minimal inadvertent adjacent tissue damage [16,17,18,19,39,41,42]. There would be minimal disruption of the Haversian system and collagen framework, improving tissue healing responses for osseointegration. The laser power levels and appropriate air–water coolant are essential during laser-assisted osteotomy to prevent thermal damage in surrounding tissues. Lasers with high absorption coefficients for water and hydroxyapatite such as titanium:sapphire (800 nm), erbium, chromium:yttrium, scandium, gallium, garnet (Er, Cr:YSGG, 2780 nm), erbium:yttrium, aluminum, garnet (Er:YAG, 2940 nm), and carbon dioxide (CO_2_, 9600 nm) lasers provide optimal precision and depth control [17,32,37,41,42,43,44,45,46]. Further, utilizing the newer femtosecond lasers capable of ultrashort pulses with high incident energy and an optimal transmission period shorter than the thermal tissue relaxation times would achieve ideal surgical outcomes. Additional hemostasis (photocoagulation) and surgical site disinfection, as noted above, would be expected to further improve implant–biological tissue interactions [19,37]. We also identified a few specific limitations of the laser osteotomy procedures, such as increased auditory disruptions (cracking sounds) which may cause discomfort for the patient and practitioner [47]. Other limitations included the high cost of the equipment, the need for additional safety with invisible infrared wavelength lasers, and advanced training requirements that emphasize a thorough understanding of power and parameter modifications.

A prior publication describes a manually operated laser system for bone grafts [48]. It cites several discrete advantages of the laser approach in its ability to avoid osteonecrosis and micro-cracks due to it precise, depth-controlled, atraumatic technique compared with routine mechanical osteotomy. The ability to generate an osteotomy with various shapes is feasible with a laser system but is limited with rotary cutting systems. This study noted that the initial stability and bone–implant contact (BIC) at 90 days post-surgery with the laser osteotomy was similar to mechanical osteotomy [37]. Automation of this procedure could add significant advantages such as precision navigation, ease and convenience of use, active sensing to distinguish between soft and hard tissues to cease laser operation, and improved overall efficacy with minimal human intervention [42,49].

## 9. Barriers to Automation in Clinical Dentistry

Surgical procedures conducted autonomously hold various benefits, such as offering medical assistance in remote areas with little to no access to transportation, in conflict zones, and during space travel, where access to dentists or surgeons will be limited. It can also serve as a valuable tool in routine high-volume, clinical scenarios to ensure consistent quality care for all. It can also, theoretically, reduce cost and, hence, improve access to care. Despite these advantages, we identified several obstacles to the practical implementation of autonomous surgical robotics. First, patient acceptance poses a significant obstacle that may impede the widespread adoption of fully autonomous surgical robots. As with any new technology, these robotic systems must establish their safety before gaining trust and recognition in the medical field. Second, to ensure patient safety, regulatory agencies have established stringent guidelines prior to their clinical use. These regulatory clearances, along with the high liability in healthcare procedures, add cost and time to practical clinical adoption. From the clinician’s perspective, these regulatory burdens would naturally entail a larger financial barrier to acquiring and operating these devices. Additionally, dentists will need to undergo specialized training on how to properly and safely operate these technologies, with the ability to intervene when necessary. A more existential aspect is the prominence of traditional training and years of clinical practice that hinder the adoption of new technologies, especially for complex operations with reduced or minimal control. It is evident that some unlearning and discomfort are essential components of adopting novel technologies. Ultimately, implementing these innovative technologies must accomplish an improved level of professional practice and patient satisfaction. 

There are also several misconceptions and apprehensions of autonomous systems undermining job security that need to be addressed at the societal and policy levels. In order for the technology to be deployed successfully, it is important to acknowledge that these are early phases of development and there may be failures or limitations that could be iteratively improved [50]. Interestingly, a recent survey investigated patients’ propensity to embrace automated surgery for simple procedures that noted men were more predisposed than women in accepting this option [51]. However, both genders had a lower acceptance as the complexity and invasiveness of the surgical procedures increased. Such findings underscore gender differences in attitudes towards novel technological innovations as well as highlight the importance of technical intricacies while introducing new healthcare services [51]. A positive outlook is critical for patients and clinicians in order to increase the pace of adoption of these technologies.

Finally, another critical factor affecting adoption of a new technology is its financial impact. The upfront investment to procure equipment and training as well as the developmental costs of maintaining and successfully utilizing these technologies are significant considerations. It stands to reason that as the user base grows, economies of scale may allow for greater availability and reduced costs. A technologically advanced system that has significantly improved precision and improves clinical outcomes will have better patient acceptance and eventually overcome all the necessary barriers for widespread acceptance. 

## 10. Conclusions and Future Directions

The future of automation using robotics and precision-engineered devices in implant and clinical dentistry appears to be poised to gain from two discrete, major domains of general technological advances, namely, the software and the hardware industries. The advances in massively parallel processing, quantum computers, and laser processing units have enabled phenomenal advances in software with machine learning and artificial intelligence (AI). Digitation of healthcare presents immense opportunities and challenges on the software front. The global data total in 2021 was over 64.2 zettabytes, of which over 30% came from the healthcare industry. This vast amount of data has allowed the biomedical community to apply artificial intelligence (AI) and machine learning (ML) techniques in areas such as predictive analysis, virtual diagnosis, and patient monitoring.

**Figure 6:**
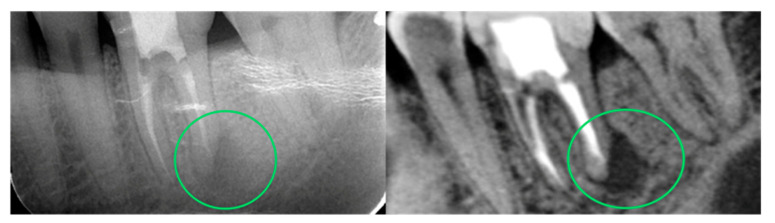
A comparison of conventional radiographs (**left**) and automated AI-enhanced radiographs (**right**) discretely outlying the anatomical features that can aid in improved diagnosis, prognosis, and accurate follow-up care. The highlighted area (green circle) show diagnostic enhanced region of interest.

A simple and current application showcasing AI in routine clinical dentistry is the automated image enhancements from cone-beam computed tomography (CBCT) imaging allowing key anatomical structures to be distinguished (Figure 6) [52]. There have been a few studies conducted that have implemented AI and ML models in implantology. One such study conducted by Li et al. developed a model using finite element analysis (FEA) capable of measuring the stress at the implant–bone interface by considering implant length, implant thread length, and thread pitch [53]. FEA uses calculations, models, and simulations to predict and understand the mechanical behavior of objects under various physical conditions. In the case of implant dentistry, the focus of these researchers was on the stress concentration of the implant. Another group used deep learning (DL) as a means to predict implant outcome (success or failure) from peripheral and panoramic films [54]. They divided 248 patients (89 with failed implants and 159 with successful implants) into three categories (implant failure with marginal bone loss, implant failure without marginal bone loss, and implant success). Using 529 peripheral and 551 panoramic images, they trained a convolution neural network (CNN) to extract key features from their data and built a hybrid model that combined the two types of images to predict the outcome of future implants.

Although these techniques have huge potential, it is important to continue research in these areas in order to lay the foundation for effective future clinical integration. There are several factors that were not considered by Li et al.’s model for stress concentration and vice versa with Zhang et al.’s model for implant-outcome prediction. It is important to consider many direct or indirect aspects of the implant procedure. This is because identifying causality can majorly influence the predictive outcome of an implant’s clinical success. In other words, increasing the amount of available training data and identifying the most significant feature influencing the outcome, we can try to uncover the true causal relationship and create a more accurate prediction model. This not only increases precision and quality of care, but also impacts the implant long-term functional success rate rather than simply the success or failure of the immediate implant placement. These multivariate, large dataset analyses directly lend themselves to AI to overcome the shortcomings of traditional clinical dental care, which has been widely criticized.

Another discrete aspect of dental care is the anxiety and fear associated with seeking dental care. In addition to the clinical procedures involving sharp surgical instruments and mechanical vibrations, barriers to care also include accessibility and pricing. Dentists, on the other hand, face a distinct set of difficulties, such as ergonomics, human error, lack of tactile feedback, shifting social norms around the use of lasers rather than traditional drills, and weariness. Over time, therapies have evolved, but problems with treatments, such as poor visibility, inconsistent surface quality, restricted precision, and restricted access, still exist. A flexible technology used effectively in precise industrial micromachining of metals, ceramics, polymers, glass, and biological material for medical usage in eye surgery is ultra-fast femtosecond (1 fs = 10–15 s) laser micromachining [55,56,57]. By combining existing technology with future developments intended to advance dentistry, this field will reach new heights. Automation reduces the physical and emotional strain of the workplace, preventing dentists from becoming physically and mentally burned out while also boosting their output.

In summary, automation in implant osteotomy offers a versatile approach to treating conditions like resorbed bone and for sinus augmentation, zygomatic-implant cases, autogenic and allogenic shell techniques for alveolar ridge augmentation, and bone enhancement procedures. The complexity of these individual clinical scenarios would appear to significantly benefit from the precision, minimal operating time, procedural cost, and patient comfort of using the automation approaches. The impact of automated robotics could be generalized, well beyond simply placing implants, to other routine and sophisticated surgical techniques. Further, although current literature is limited, the utility of lasers for various implant procedures offers significant new avenues for increased precision and refinement of these automated approaches. Overall, these areas hold great promise for the future of clinical dentistry for both routine and low-resource rural settings and for space travel, where it offers a sustainable, precise, and effective approach.

## Data Availability

No new data were created in this work.

## References

[1] Elagib M.F.A., Alqaysi M.A.H., Almushayt M.O.S., Nagate R.R., Gokhale S., Chaturvedi S. (2022). Dental implants in growing patients: A systematic review and meta-analysis. Technol. Health Care.

[2] Tyrovolas S., Koyanagi A., Panagiotakos D.B., Haro J.M., Kassebaum N.J., Chrepa V., Kotsakis G.A. (2016). Population prevalence of edentulism and its association with depression and self-rated health. Sci. Rep..

[3] Venkat M., Janakiram C., Ramanarayanan V. (2021). Prevalence of Tooth Mortality among Adults in India: A Systematic Review and Meta-Analysis. Contemp. Clin. Dent..

[4] Zelig R., Goldstein S., Touger-Decker R., Firestone E., Golden A., Johnson Z., Kaseta A., Sackey J., Tomesko J., Parrott J.S. (2022). Tooth Loss and Nutritional Status in Older Adults: A Systematic Review and Meta-analysis. JDR Clin. Trans. Res..

[5] Aiquel L.L., Pitta J., Antonoglou G.N., Mischak I., Sailer I., Payer M. (2021). Does the timing of implant placement and loading influence biological outcomes of implant-supported multiple-unit fixed dental prosthesis-A systematic review with meta-analyses. Clin. Oral Implant. Res..

[6] Berlin-Broner Y., Levin L. (2020). Dental Implant Success and Endodontic Condition of Adjacent Teeth: A Systematic Review. Int. J. Oral Maxillofac. Implant..

[7] Hamed M.T., Mously H.A., Ghulman M.M., Naguib G.H. (2021). Impact of dental implant diameter on the efficiency of fatigue: A systematic review analysis. J. Pak. Med. Assoc..

[8] Guazzi P., Grandi T., Grandi G. (2015). Implant site preparation using a single bur versus multiple drilling steps: 4-month post-loading results of a multicenter randomised controlled trial. Eur. J. Oral Implant..

[9] Arcuri L., Lorenzi C., Cecchetti F., Germano F., Spuntarelli M., Barlattani A. (2015). Full digital workflow for implant-prosthetic rehabilitations: A case report. Oral Implant..

[10] Bolding S.L., Reebye U.N. (2022). Accuracy of haptic robotic guidance of dental implant surgery for completely edentulous arches. J. Prosthet. Dent..

[11] Cheng K.J., Kan T.S., Liu Y.F., Zhu W.D., Zhu F.D., Wang W.B., Jiang X.F., Dong X.T. (2021). Accuracy of dental implant surgery with robotic position feedback and registration algorithm: An in-vitro study. Comput. Biol. Med..

[12] Kan T.S., Cheng K.J., Liu Y.F., Wang R., Zhu W.D., Zhu F.D., Jiang X.F., Dong X.T. (2022). Evaluation of a custom-designed human-robot collaboration control system for dental implant robot. Int. J. Med. Robot..

[13] Yang S., Chen J., Li A., Li P., Xu S. (2022). Autonomous Robotic Surgery for Immediately Loaded Implant-Supported Maxillary Full-Arch Prosthesis: A Case Report. J. Clin. Med..

[14] Tack P., Victor J., Gemmel P., Annemans L. (2016). 3D-printing techniques in a medical setting: A systematic literature review. Biomed. Eng. Online.

[15] Yan B., Zhang W., Cai L., Zheng L., Bao K., Rao Y., Yang L., Ye W., Guan P., Yang W. (2022). Optics-guided Robotic System for Dental Implant Surgery. Chin. J. Mech. Eng..

[16] Ashforth S.A., Oosterbeek R.N., Bodley O.L.C., Mohr C., Aguergaray C., Simpson M.C. (2020). Femtosecond lasers for high-precision orthopedic surgery. Lasers Med. Sci..

[17] Rajitha Gunaratne G.D., Khan R., Fick D., Robertson B., Dahotre N., Ironside C. (2017). A review of the physiological and histological effects of laser osteotomy. J. Med. Eng. Technol..

[18] Stubinger S. (2010). Advances in bone surgery: The Er:YAG laser in oral surgery and implant dentistry. Clin. Cosmet. Investig. Dent..

[19] Pantawane M., Dahotre N. (2019). Challenges and Advances in Osteotomy. Ann. Bone Jt. Surg..

[20] Gulati M., Anand V., Salaria S.K., Jain N., Gupta S. (2015). Computerized implant-dentistry: Advances toward automation. J. Indian Soc. Periodontol..

[21] Wu Y., Wang F., Fan S., Chow J.K. (2019). Robotics in Dental Implantology. Oral Maxillofac. Surg. Clin. N. Am..

[22] Soundaria Saravanan C.A.M., Singaravelu S.K., Raju P.K. (2020). Application of digital technology in implant dentistry-an overview. Int. J. Adv. Res..

[23] Spielau T., Hauschild U., Katsoulis J. (2019). Computer-assisted, template-guided immediate implant placement and loading in the mandible: A case report. BMC Oral Health.

[24] Marques S., Ribeiro P., Falcao C., Lemos B.F., Rios-Carrasco B., Rios-Santos J.V., Herrero-Climent M. (2021). Digital Impressions in Implant Dentistry: A Literature Review. Int. J. Environ. Res. Public. Health.

[25] Tanna N.K., AlMuzaini A., Mupparapu M. (2021). Imaging in Orthodontics. Dent. Clin. N. Am..

[26] Vafaee F., Firouz F., Mohajeri M., Hashemi R., Ghorbani Gholiabad S. (2021). In vitro Comparison of the Accuracy (Precision and Trueness) of Seven Dental Scanners. J. Dent..

[27] Verykokou S., Ioannidis C. (2023). An Overview on Image-Based and Scanner-Based 3D Modeling Technologies. Sensors.

[28] Kernen F., Kramer J., Wanner L., Wismeijer D., Nelson K., Flugge T. (2020). A review of virtual planning software for guided implant surgery—data import and visualization, drill guide design and manufacturing. BMC Oral Health.

[29] Vasoglou G., Stefanidaki I., Apostolopoulos K., Fotakidou E., Vasoglou M. (2022). Accuracy of Mini-Implant Placement Using a Computer-Aided Designed Surgical Guide, with Information of Intraoral Scan and the Use of a Cone-Beam CT. Dent. J..

[30] Tahmaseb A., Wu V., Wismeijer D., Coucke W., Evans C. (2018). The accuracy of static computer-aided implant surgery: A systematic review and meta-analysis. Clin. Oral. Implant. Res..

[31] Aydemir C.A., Arisan V. (2020). Accuracy of dental implant placement via dynamic navigation or the freehand method: A split-mouth randomized controlled clinical trial. Clin. Oral Implant. Res..

[32] Roessler K., Winter F., Wilken T., Pataraia E., Mueller-Gerbl M., Dorfer C. (2021). Robotic Navigated Laser Craniotomy for Depth Electrode Implantation in Epilepsy Surgery: A Cadaver Lab Study. J. Neurol. Surg. A Cent. Eur. Neurosurg..

[33] Talwar N., Chand P., Singh B.P., Rao J., Pal U.S., Ram H. (2012). Evaluation of the efficacy of a prosthodontic stent in determining the position of dental implants. J. Prosthodont..

[34] Tao B., Feng Y., Fan X., Zhuang M., Chen X., Wang F., Wu Y. (2022). Accuracy of dental implant surgery using dynamic navigation and robotic systems: An in vitro study. J. Dent..

[35] Houssiau F.A., Devogelaer J.P., Van Damme J., de Deuxchaisnes C.N., Van Snick J. (1988). Interleukin-6 in synovial fluid and serum of patients with rheumatoid arthritis and other inflammatory arthritides. Arthritis Rheum..

[36] Ahmad P., Alam M.K., Aldajani A., Alahmari A., Alanazi A., Stoddart M., Sghaireen M.G. (2021). Dental Robotics: A Disruptive Technology. Sensors.

[37] Apostolopoulos A.P., Angelis S., Kaitatzi M., Kareliotis G., Tsiotsias A., Maris S.J., Filippou D.K., Makropoulou M. (2021). The Facts and Myths for the Use of Lasers in Orthopedic Surgery. J. Long. Term. Eff. Med. Implant..

[38] Romanos G.E., Gupta B., Yunker M., Romanos E.B., Malmstrom H. (2013). Lasers use in dental implantology. Implant Dent..

[39] Xue V.W., Zhao I.S., Yin I.X., Niu J.Y., Lo E.C.M., Chu C.H. (2021). Effects of 9300 nm Carbon Dioxide Laser on Dental Hard Tissue: A Concise Review. Clin. Cosmet. Investig. Dent..

[40] Heyder M., Sigusch B., Hoder-Przyrembel C., Schuetze J., Kranz S., Reise M. (2022). Clinical effects of laser-based cavity preparation on class V resin-composite fillings. PLoS ONE.

[41] Domke M., Wick S., Laible M., Rapp S., Huber H.P., Sroka R. (2018). Ultrafast dynamics of hard tissue ablation using femtosecond-lasers. J. Biophotonics.

[42] Abbasi H., Beltran Bernal L.M., Hamidi A., Droneau A., Canbaz F., Guzman R., Jacques S.L., Cattin P.C., Zam A. (2020). Combined Nd:YAG and Er:YAG lasers for real-time closed-loop tissue-specific laser osteotomy. Biomed. Opt. Express.

[43] Nalcaci R., Cokakoglu S. (2013). Lasers in orthodontics. Eur. J. Dent..

[44] Honigmann P., Hofer M., Hirsch S., Morawska M., Muller-Gerbl M., Thieringer F.M., Coppo E. (2022). Cold ablation robot-guided laser osteotomy in hand, wrist and forearm surgery-A feasibility study. Int. J. Med. Robot..

[45] Moslemi N., Shahnaz A., Masoumi S., Torabi S., Akbari S. (2017). Laser-Assisted Osteotomy for Implant Site Preparation: A Literature Review. Implant. Dent..

[46] Arora A., Upadhyaya V., Munjal D., Malik D., Parashar D. (2017). Lasers—A Benefaction to Implant Dentistry. IOSR J. Dent. Med. Sci..

[47] Chery J., Freitas V., Saman D.M., Gupta A. (2022). Comparison of Cavity Preparation Times Using Conventional High-Speed Handpiece Versus Er, Cr:YSGG Laser: A Pilot Study with Pediatric Dental Residents. Pediatr. Dent..

[48] Stubinger S., Landes C., Seitz O., Sader R. (2007). Er:YAG laser osteotomy for intraoral bone grafting procedures: A case series with a fiber-optic delivery system. J. Periodontol..

[49] Nguendon Kenhagho H., Canbaz F., Gomez Alvarez-Arenas T.E., Guzman R., Cattin P., Zam A. (2021). Machine Learning-Based Optoacoustic Tissue Classification Method for Laser Osteotomes Using an Air-Coupled Transducer. Lasers Surg. Med..

[50] Chan K.S., Kwan J.R., Shelat V.G. (2022). Awareness, perception, knowledge, and attitude toward robotic surgery in a general surgical outpatient clinic in Singapore, Asia. J. Clin. Transl. Res..

[51] McDermott H., Choudhury N., Lewin-Runacres M., Aemn I., Moss E. (2020). Gender differences in understanding and acceptance of robot-assisted surgery. J. Robot. Surg..

[52] Jacobs R., Salmon B., Codari M., Hassan B., Bornstein M.M. (2018). Cone beam computed tomography in implant dentistry: Recommendations for clinical use. BMC Oral Health.

[53] Li H., Shi M., Liu X., Shi Y. (2019). Uncertainty optimization of dental implant based on finite element method, global sensitivity analysis and support vector regression. Proc. Inst. Mech. Eng. Part H.

[54] Zhang C., Fan L., Zhang S., Zhao J., Gu Y. (2023). Deep learning based dental implant failure prediction from periapical and panoramic films. Quant. Imaging Med. Surg..

[55] Chen H., Li H., Sun Y., Wang Y., Lu P. (2016). Femtosecond laser for cavity preparation in enamel and dentin: Ablation efficiency related factors. Sci. Rep..

[56] Chung S.H., Mazur E. (2009). Surgical applications of femtosecond lasers. J. Biophotonics.

[57] Rapp L., Madden S., Brand J., Maximova K., Walsh L.J., Spallek H., Zuaiter O., Habeb A., Hirst T.R., Rode A.V. (2023). Investigation of laser wavelength effect on the ablation of enamel and dentin using femtosecond laser pulses. Sci. Rep..

